# Vitamin D level is associated with rupture of intracranial aneurysm in patients with subarachnoid hemorrhage

**DOI:** 10.3389/fneur.2022.890950

**Published:** 2022-08-11

**Authors:** Sen Wei, Xin Yuan, Dongdong Li, Feng Fan, Xinbin Guo, Yuming Xu, Sheng Guan

**Affiliations:** ^1^Department of Neurointervention, The First Affiliated Hospital of Zhengzhou University, Zhengzhou, China; ^2^Department of Neurology, The First Affiliated Hospital of Zhengzhou University, Zhengzhou, China

**Keywords:** intracranial aneurysm, vitamin D, subarachnoid hemorrhage, rupture, SAH

## Abstract

**Background:**

Increasing evidence supports the relationship between vitamin D levels and stroke. However, there are few studies on the association between vitamin D levels and subarachnoid hemorrhage (SAH), especially in patients with aneurysmal SAH. The present study investigated the association between vitamin D level and rupture of intracranial aneurysm in a cohort of patients with SAH.

**Methods:**

The data of patients diagnosed with SAH at our hospital between September 2019 and December 2020 were retrospectively reviewed. Patients' information was collected, and serum vitamin D levels were measured. Computed tomography was performed to confirm SAH diagnosis, and digital subtraction angiography was performed to determine whether SAH was caused by rupture of an intracranial aneurysm. Multivariate logistic regression analyses were performed to investigate the association between vitamin D level and aneurysmal SAH.

**Results:**

Overall, 193 patients with SAH were evaluated; 160 with aneurysmal SAH (age 55.86 ± 12.30 years, 63.15% female) and 33 with non-aneurysmal SAH (age 56.21 ± 9.92 years, 45.45% female). Univariate analysis showed that the vitamin D level in aneurysmal SAH was lower than that in non-aneurysmal SAH (16.95 ± 8.69 vs. 22.74 ± 9.12 ng/ml, *p* = 0.001). In addition, there were more patients with hypertension in aneurysmal SAH group than in non-aneurysmal SAH group (53.75 vs. 24.24%, *p* = 0.002). Notably, there was still a strong correlation between vitamin D level and aneurysmal SAH after adjusting for confounders in the multivariate model [OR (odds ratio), 0.935; 95% CI (confidence interval), 0.890–0.983; *p* = 0.008].

**Conclusion:**

Vitamin D level is associated with rupture of intracranial aneurysm in patients with SAH. Patients with aneurysmal SAH have lower vitamin D levels than those with non-aneurysmal SAH.

## Introduction

Subarachnoid hemorrhage (SAH) is an acute neurological emergency with an overall fatality rate of 42% at 28 days. Altogether, 85% of SAH cases are caused by rupture of intracranial aneurysms. Aneurysmal re-rupture and second bleeds are associated with a mortality rate of 70–90% (1). Therefore, early identification and treatment of intracranial aneurysms can significantly improve the survival rate of patients with SAH.

Subarachnoid hemorrhage diagnosis is relatively easy on using computed tomography (CT) and laboratory analysis, such as cerebrospinal fluid (CSF) analysis (2). To further clarify the cause of bleeding, vascular imaging, including CT angiography (CTA) and digital subtraction angiography (DSA) are required to identify the presence of aneurysms. However, sometimes, the initial imaging examination findings of patients with an aneurysmal pattern of hemorrhage are negative (3). Therefore, the identification of indicators to prompt the cause of SAH is crucial.

As a serum inflammatory marker, vitamin D has been established to be associated with acute stroke in recent research (4–6). Few studies have revealed that vitamin D level is related to the occurrence and rupture of intracranial aneurysms. However, SAH itself may be cause a series of inflammatory reactions (7) and these studies cannot rule out the effect of SAH on vitamin D levels as an inflammatory marker (8). Therefore, the present study aims to determine whether vitamin D levels are associated with aneurysmal rupture in patients with SAH.

## Methods

### Subjects

Patients with SAH were retrospectively and consecutively enrolled at the Neurointerventional Department of the First Affiliated Hospital of Zhengzhou University (Henan, China) from September 2019 to December 2020. The following inclusion criteria were applied: (1) age ≥ 18 years; (2) hospitalized with a primary diagnosis of spontaneous SAH according to World Health Organization criteria, which was confirmed through brain CT within 7 days of symptom onset; and (3) no severe physical illnesses that were life-threatening or interfering with the stroke recovery evaluation. Patients who did not undergo CT or CTA/DSA imaging and those with rare causes of SAH such as cerebrovascular malformations, trauma, etc, were excluded.

All patients with SAH underwent DSA after admission. Patients with SAH, included in the study, were divided into two groups: aneurysmal SAH and non-aneurysmal SAH. Patients with an initial negative DSA were re-examined using CTA or DSA 2 weeks after onset. Patients with a negative re-examination were re-examined using CTA 3 months after onset. Patients with aneurysms at any time point were included in the aneurysmal SAH group, and those without aneurysms at all examinations were included in the non-aneurysmal SAH group.

### Ethics statement

The study protocol was approved by the Ethics Committee of the First Affiliated Hospital of Zhengzhou University. Informed consent for relevant examination and treatment were obtained from all patients or their legally authorized representatives.

### Data collection

Detailed baseline data were collected, including age, sex, hypertension, coronary artery disease (CAD), stroke, hyperlipidemia, diabetes mellitus, and medical history. Hypertension was defined according to the 2017 ACC/AHA/AAPA/ABC/ACPM/AGS/APhA/ASH/ASPC/NMA/PCNA Guidelines for the Prevention, Detection, Evaluation, and Management of High Blood Pressure in Adults: Executive Summary (9). Hypercholesterolemia and diabetes mellitus were defined according to the 2018 AHA/ACC/AACVPR/AAPA/ABC/ACPM/ADA/AGS/AphA/ASPC/NLA/PCNA Guidelines on the Management of Blood Cholesterol (10), and the American Diabetes Association criteria, respectively (11). Stroke was defined as a history of stroke. CAD was defined as a history of angina pectoris, myocardial infarction, or use of medication to control CAD. Smoking was defined as ≥10 cigarettes per day for >3 months; Drinking of alcohol was considered if it occurred at least once per week (≥ 500 ml) for more than 3 months.

### Laboratory examination

Fasting blood samples were collected after an overnight fasting of at least 8 h for the measurement of serum calcium, phosphorus, creatinine, uric acid, cholesterol, and vitamin D levels on the morning of day 1 post admission.

Estimated glomerular filtration rate (eGFR), an estimate of the baseline kidney function, was calculated using the Modification of Diet in Renal Disease formula (12).

#### Vitamin D measurements

Quantitative determination of total 25-hydroxy (25-OH) vitamin D was performed using the electrochemical luminescence assay method on an e-602 Hitachi Roche Modular system machine (Roche Diagnostics, Cobas e602/2010/Japan, Basel, Switzerland).

### Statistical analyzes

Statistical analysis was performed using SPSS software (version 23.0; SPSS, Inc. IBM Corp., Armonk, NY, USA). Numerical variables are described as mean ± standard deviation, and assessed with Student's *t*-test for independent samples. When the normality assumption was violated, Mann–Whitney *U*-test was used. Categorical variables were described by grouped data and percentages, and rates or percentages between the groups were compared using the χ^2^-test or Fisher's exact test. All factors associated with aneurysmal SAH from the univariate analyses at a threshold (*p* < 0.1) and age were included in the multivariate model as candidate variables and then removed by a forward selection procedure. Two-tailed tests with a probability (*p* < 0.05), were used to estimate statistical significance for multivariate logistic regression analyzes.

## Results

### Study population and baseline characteristics

**Figure 1:**
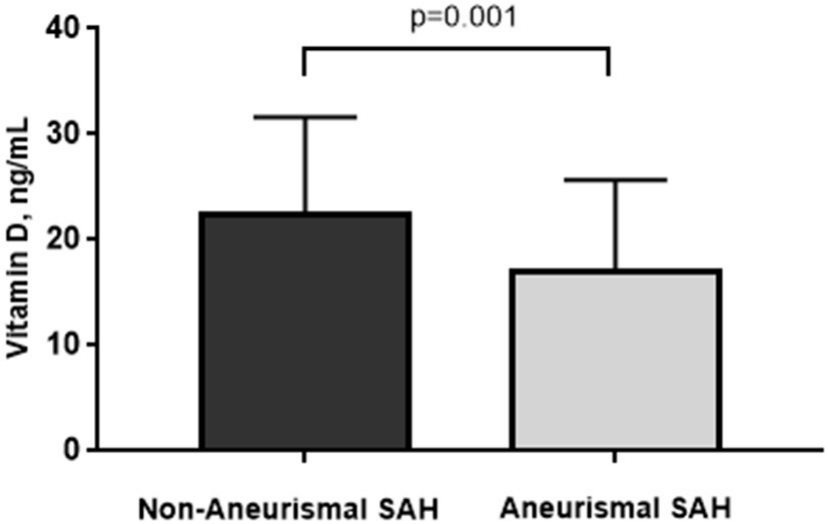
Vitamin D levels were significantly lower in the aneurysmal SAH group than in the non-aneurysmal SAH group (16.95 ± 8.69 vs. 22.74 ± 9.12 ng/ml, *p* = 0.001).

A total of 193 patients with SAH were analyzed. There were 160 patients with aneurysmal SAH, with a mean age of 55.86 ± 12.30 years and 63.15% females, and 33 patients with non-aneurysmal SAH, with a mean age of 56.21 ± 9.92 years and 45.45% females. There was no significant difference in age and sex between the two groups. In the univariate analysis, vitamin D levels were significantly lower in the aneurysmal SAH group than in the non-aneurysmal SAH group (16.95 ± 8.69 vs. 22.74 ± 9.12 ng/ml, *p* = 0.001; Figure 1). There were more patients with hypertension in the aneurysmal SAH group than in the non-aneurysmal SAH group (53.75 vs. 24.24%, *p* = 0.002). There were no significant differences in CAD, stroke, hypercholesterolemia, diabetes mellitus, drinking, smoking, aspirin use, or other laboratory examinations (homocysteine, eGFR, serum calcium, and phosphorus levels) (all *p* > 0.1). Detailed baseline characteristics and the results of the univariate analysis are shown in Table 1.

**Table 1:** Baseline characteristics and univariate determinants of the patients with SAH.

**Index**	**Aneurismal SAH** **(*n* = 160)**	**Non-aneurismal SAH** **(*n* = 33)**	***P*** **-value**
Age, year	55.86 ± 12.30	56.21 ± 9.92	0.878
**Sex, n (%)**			0.059
Female	101 (63.13)	15 (45.45)	
Male	59 (36.87)	18 (54.55)	
Hypertension, n (%)	86 (53.75)	8 (24.24)	0.002
CAD, n (%)	15 (9.38)	1 (3.03)	0.229
Stroke, n (%)	21 (13.13)	2 (6.06)	0.254
Hypercholesterolemia, n (%)	42 (26.25)	9 (27.27)	0.905
Diabetes mellitus, n (%)	59 (36.88)	9 (27.27)	0.505
Aspirin use, n (%)	23 (14.38)	8 (24.24)	0.160
Smoking, n (%)	30 (18.75)	8 (24.24)	0.470
Drinking, n (%)	21 (13.13)	6 (18.18)	0.403
Homocysteine, umol/	16.93 ± 8.24	14.98 ± 5.11	0.216
eGFR^*^	116.24 ± 26.06	119.05 ± 27.58	0.577
Vitamin D, ng/ml	16.95 ± 8.69	22.74 ± 9.12	0.001
Ca, mmol/L	2.29 ± 0.23	2.26 ± 0.10	0.517
P, mmol/L	1.02 ± 0.32	1.02 ± 0.20	0.970

* *Estimated glomerular filtration rate (eGFR), an indicator of baseline kidney function, was calculated with the Modification of Diet in Renal Disease formula. eGFR = 175 x (SCr)^−1.154^ x (age)^−0.203^ x 0.742 [if female]. SAH, subarachnoid hemorrhage; CAD, Coronary artery disease; eGFR, estimated glomerular filtration rate; Ca, calcium; P, phosphorus*.

In the multivariate logistic regression analysis, the association between vitamin D level and aneurysmal SAH was still significant (OR, 0.935; 95% CI: 0.890–0.983, *p* = 0.008) after adjusting for age, sex and hypertension. In addition, hypertension was independently associated with aneurysmal SAH (OR: 4.256; 95% CI: 1.703–10.635, *p* = 0.002; Table 2).

**Table 2:** Multivariate logistic regression analysis between patients with aneurysmal SAH and non-aneurysmal SAH.

**Index**	**OR**	**95% CI**	***P*** **-value**
Age, year	0.996	0.961–1.032	0.827
Female sex, %	0.803	0.316–2.041	0.644
Hypertension, %	4.256	1.703–10.635	0.002
Vitamin D, ng/ml	0.935	0.890–0.983	0.008

## Discussion

The study shows that vitamin D level is associated with rupture of intracranial aneurysm in patients with SAH. Vitamin D levels were significantly lower in patients with aneurysmal SAH than in those with non-aneurysmal SAH.

The relationship between vitamin D and the cerebral aneurysms was first proposed by Guan et al. (13). They found that vitamin D levels were lower in patients with cerebral aneurysms than in those without it, which was also verified by the later observational study of small samples. These studies suggest that vitamin D may be related to the occurrence of intracranial aneurysms. Our study found that vitamin D levels were independently associated with rupture of intracranial aneurysms in patients with SAH, which was somewhat consistent with results from the previous studies. To our knowledge, there are few studies dedicated to the association between vitamin D and intracranial aneurysms in patients with SAH.

The mechanism of vitamin D in the occurrence of aneurysms is still unclear. We elaborated on the possible specific mechanisms. Previous studies suggested that both structural fragility and aneurysm growth might be mediated by aneurysmal wall inflammation, which was verified by vessel wall magnetic resonance imaging studies through aneurysmal wall enhancement (14, 15) and vitamin D may be involved as an inflammatory marker. In our previous study, we found that vitamin D levels were lower in patients with ruptured intracranial aneurysms than those with unruptured intracranial aneurysms. However, we cannot completely rule out the influence of SAH itself on laboratory indicators. In the study, both univariate and multivariate analysis suggested that there was a strong correlation between vitamin D levels and rupture of aneurysms in patients with SAH. Therefore, we concluded that vitamin D levels may be related to the rupture of intracranial aneurysms, not the subsequent decrease of SAH based on our present study and previous study. The mechanism of vitamin D in the rupture of intracranial aneurysms needs further research in the future.

In addition, the study also observed that hypertension was associated with aneurysmal SAH, which has been identified in epidemiological studies. In a prospective population-based cohort, hypertension has been shown to be one of the most important risk factors for aneurysmal SAH (16). An animal model of aneurysm can be induced by a combination of hypertension and disruption of collagen synthesis (17). Researchers believe that the hemodynamic stress at the bifurcation site of the cerebral arteries and mechanical damage in the arterial wall caused by hypertension are the potential factors of intracranial aneurysms (18).

The strengths of our study are as follows. Firstly, to the best of our knowledge, this study is the first research to explore the relationship between vitamin D levels and aneurysmal rupture in patients with SAH. Secondly, a relatively large sample size of patients with SAH was included in the present study. The large sample size provided a considerable statistical power, which contributed to detect real differences of vitamin D level among patients with aneurysmal SAH and in those with non-aneurysmal SAH. Thirdly, in the non-aneurysmal SAH group, intracranial aneurysms or other rare causes such as cerebrovascular malformations were excluded by CTA or DSA repeatedly; therefore, our grouping conditions were strict and clear. Moreover, this study includes many factors related to vitamin D, such as calcium and phosphorus level, renal function and so on in the univariate analyses. All factors associated with aneurysmal SAH from the univariate analyses at a threshold (*p* < 0.1), especially sex, which was found to be associated with intracranial aneurysms and vitamin D levels, were included in the multivariate analyses in order to eliminate the interference of other factors. The conclusion is relatively convincing.

Of course, the study also had several limitations. First, the current study utilized a cross-sectional design; thus, a causal relationship between vitamin D levels and the aneurysmal SAH needs further confirmation using a longitudinal study. Second, the study was a single-center retrospective study, which inevitably resulted in selection bias. These results may be related to the patient group. In addition, owing to the small number of patients in the non-aneurysm group, subgroup analysis of patients with different causes was not possible.

## Conclusion

Our study showed that vitamin D levels are associated with rupture of intracranial aneurysms in patients with SAH; patients with aneurysmal SAH have lower vitamin D levels than those with non-aneurysmal SAH. However, further research is needed to verify the relationship and clarify the mechanisms underlying these associations.

## Data availability statement

The original contributions presented in the study are included in the article/supplementary material, further inquiries can be directed to the corresponding authors.

## Ethics statement

The studies involving human participants were reviewed and approved by Ethics Committee of the First Affiliated Hospital of Zhengzhou University. The patients/participants provided their written informed consent to participate in this study.

## Author contributions

SW designed the whole study, collected, and analyzed the data. XY drafted the manuscript. DL and FF critically reviewed the manuscript for important intellectual content. XG discussed and contributed to the analysis of the data. YX and SG critically reviewed the manuscript for important intellectual content. All authors contributed to the article and approved the submitted version.

## Conflict of interest

The authors declare that the research was conducted in the absence of any commercial or financial relationships that could be construed as a potential conflict of interest.

## Publishers Note

All claims expressed in this article are solely those of the authors and do not necessarily represent those of their affiliated organizations, or those of the publisher, the editors and the reviewers. Any product that may be evaluated in this article, or claim that may be made by its manufacturer, is not guaranteed or endorsed by the publisher.
